# Self-Generated Expectations of Hazard Prevalence Affect Virtual Search and Rescue

**DOI:** 10.1177/00187208251410492

**Published:** 2026-01-06

**Authors:** Yan Shan Tai, Jacques A. Grange, Robert C. Honey

**Affiliations:** 1 Cardiff University, UK

**Keywords:** self-generated expectations, instructed expectations, visual search, target prevalence, simulated emergencies

## Abstract

**Objective:**

To understand how prior expectations and instructions about hazard prevalence affect high-stakes visual search in a semi-immersive virtual environment, where participants take on the role of firefighters in search and rescue missions.

**Background:**

Information about target prevalence influences visual search in standard laboratory studies. However, little is known about how prior expectations and new information about target prevalence interact in simulated emergency scenarios.

**Methods:**

Participants (*n* = 48) received training where the average number of hazards (explosive cylinders) amongst similar distractors was varied (two or six) before participants rescued a trapped person. Trial-end feedback indicated whether all targets were removed and the person rescued. They were then instructed that hazard prevalence would increase, decrease, or stay similar during test blocks. Stress was manipulated by an ongoing alarm, the threat of trial-ending explosions, and reduced movement speed. Search performance was measured by the number and type of stimuli removed and stress was assessed using self-report and physiological measures.

**Results:**

Across high and low stress conditions, more hazards were removed and more false positives occurred (i.e., more distractors removed) when test prevalence was lower than during training, compared to when prevalence levels remained similar. False negative errors were consistently low across conditions.

**Conclusion:**

Acquired hazard expectations can override explicit instructions, leading to persistent search errors, likely due to difficulties in adjusting decision criteria.

**Application:**

These results suggest that training in high-stakes hazard search should incorporate the use of tools and techniques to help mitigate the persistent influence of outdated expectations on search performance.

## Introduction

In time-sensitive scenarios, such as search and rescue missions, misidentifying an innocuous item (a distractor) as an explosive hazard (a target) diverts attention from and delays response to the immediate threat (i.e., the explosive hazard). Aside from missing targets that are infrequent (Wolfe et al., 2005, 2007), search errors could also arise from mismatches between expected and true target prevalence. In accuracy-critical tasks, expectations about target prevalence affect visual search differently depending on whether they were based on experience (i.e., self-generated) or instructions (Ishibashi et al., 2012; Lau & Huang, 2010). However, how these factors interact when both speed and accuracy are required under stress (e.g., in search and rescue missions) remain underexplored.

The low prevalence effect (LPE) describes the robust finding that infrequent targets are more likely to be missed (Wolfe et al., 2005, 2007), possibly due to conservative perceptual decision thresholds during stimulus evaluation (Wolfe et al., 2007; Wolfe & Van Wert, 2010) and premature search termination on target-absent trials (Rich et al., 2008). When evaluating a stimulus, high prevalence shifts the starting point of evidence accumulation towards the “target” decision boundary, and low prevalence shifts it towards the “distractor” boundary (Peltier & Becker, 2016). Thus, “target” decisions are less conservative under high prevalence than under low prevalence conditions (Peltier & Becker, 2016; Wolfe & Van Wert, 2010).

### Prevalence Expectations and Instructed Expectations

Laboratory experiments manipulating target frequency in x-ray images of baggage (e.g., where the search is for weapons) or mammograms (e.g., where the search is for malignant tumors) beyond real-world rates attenuated the LPE in expert participants (Evans et al., 2011, 2013; Nakashima et al., 2015; Wolfe et al., 2013). This finding highlights the role of direct experience with visual search in generating and modifying highly established prevalence expectations among experts.

Direct instructions can also shape prevalence expectation to produce prevalence effects (Rich et al., 2008; Taylor et al., 2022; Zhang & Houpt, 2020). When informed that targets would be rare in ‘T among L’ searches, the LPE was observed (Rich et al., 2008; Zhang & Houpt, 2020), even after extended exposure to the search array (Rich et al., 2008). Subtle differences in search instructions, such as whether trials could have “0, 1, or 2 targets” versus “1 or 2 targets,” can shorten searches and reduce hit and false alarm rates (Cox et al., 2021). Beyond prevalence expectations, instructions emphasizing speed or accuracy also modulate speed-accuracy trade-off during search (Lawrence et al., 2023; McCarley, 2009): When accuracy was prioritized, saccade frequencies increased and correlated with longer reaction times, which along with enhanced target detection in the visual periphery improved hit rates (McCarley, 2009).

While instructed prevalence has a strong influence, self-generated expectations of target prevalence have a greater impact (Ishibashi et al., 2012; Lau & Huang, 2010). Self-generated expectations may develop after as few as ten target exposures and begin to reliably shape search performance (Hon & Jabar, 2018). These expectations can be overridden when individuals search under different prevalence conditions (Evans et al., 2011, 2013; Nakashima et al., 2015; Wolfe et al., 2013). However, recent evidence suggests that adaptation to changing prevalence might require more exposure than previously suggested, regardless of domain expertise (e.g., in medical image interpretation; Wolfe, 2022). In comparison, instructed prevalence seems less influential: Participants who initially experienced low target prevalence searched longer during target-absent trials within blocks described as high prevalence but still showed elevated miss error rates (Lau & Huang, 2010). Nevertheless, despite experiencing the same global prevalence rate, a high prevalence cue slightly increased false positives compared to a low prevalence cue (Ishibashi et al., 2012), and an “extremely low prevalence” cue (e.g., 3%) reduced false alarm rates and increased miss rates (Ishibashi & Kita, 2014). Thus, while under calm laboratory conditions direct experience can recalibrate prevalence expectations, in real-life emergencies individuals must rely on communicated prevalence rather than firsthand experience to adjust their expectations. For example, firefighters might be dispatched to rescue trapped people in a burning warehouse that historically stored gas cylinders, but receive new information that after a change of ownership, most, but not all, of the cylinders have been removed. How such new information affects their visual search is unknown.

The broader literature shows greater reliance on self-generated than instructed expectations in other visual tasks, which has been taken to suggest that self-generated expectations may recruit cognitive resources: They engage greater attentional and preparatory processes compared to instructed expectations (Gaschler et al., 2014; Jiang et al., 2018; Kemper et al., 2012, 2017; Oberauer et al., 2013; Schwager et al., 2017; Umbach et al., 2012). Neurophysiological evidence also suggests that self-generated expectations involve greater top-down attentional engagement and preparatory processes, possibly making their violations more demanding to process than violations of instructed expectations (Kemper et al., 2012). Thus, the influence of self-generated expectations in visual tasks might potentially be sensitive to the availability of cognitive resources, which are often depleted under stressful conditions (Hyun et al., 2019; Qin et al., 2009; Shields et al., 2016). Whether stress shifts reliance between self-generated and instructed expectations during visual search is unknown.

### Stress and Visual Search

Stress arises when perceived demands exceed coping resources (Lazarus & Folkman, 1984) and impairs working memory, cognitive flexibility, and cognitive control (Diamond, 2013; Hyun et al., 2019; Plessow et al., 2011; Shields et al., 2016). These effects are often attributed to the reallocation of cognitive resources that normally support these executive functions to managing stressors (e.g., by inhibiting stress-related interference; Hyun et al., 2019; Qin et al., 2009; Shields et al., 2016), potentially reducing reliance on anticipatory strategies in favor of less demanding reactive strategies (Steinhauser et al., 2007). Time pressure reduces search accuracy, increasing miss rates and false positives (Rieger et al., 2021; Rieger & Manzey, 2024), suggesting that visual search becomes less thorough when cognitive resources (e.g., time for evidence accumulation) are constrained. Certified screeners performed better at baggage screening tasks and perceived less time pressure and fatigue than undergraduate students performing the same task, suggesting that experience enhances search efficiency and stress perception (Chavaillaz et al., 2019). Nevertheless, it is not known how stress interacts with self-generated and instructed expectations (Ishibashi et al., 2012; Lau & Huang, 2010) during visual search.

**Figure 1.**
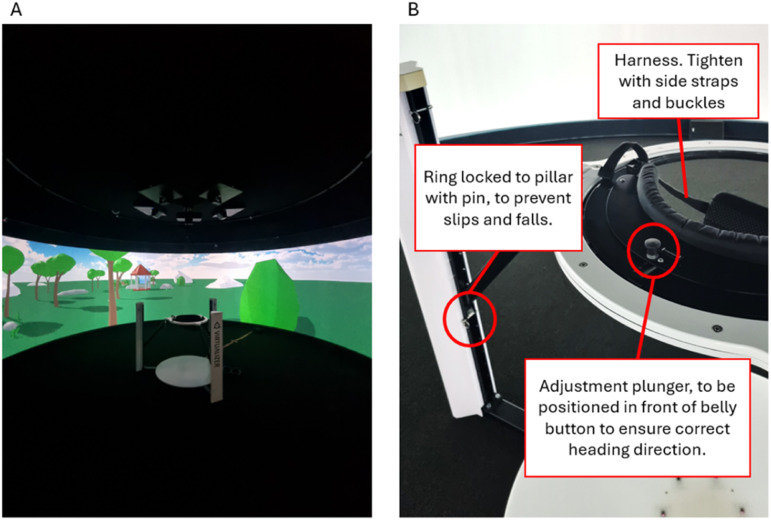
Interior of the Igloo immersive cylinder (a) and the Cyberith Virtualiser R&D kit (b).

To the best of our knowledge, the use of self-generated versus instructed expectations about target prevalence in speeded, high-stakes visual search contexts is unexplored. Virtual reality (VR) has been used in visual search research (Beitner et al., 2024; Bennett et al., 2021; Botch et al., 2023) and its adoption enables systematic manipulation of the environment to increase immersion without endangering participants (e.g., Feder et al., 2024). The present study used a semi-immersive virtual environment (VE) platform (which does not fully encompass participants’ field of vision; see Figure 1(a)) to implement a hazard removal task, where participants acted as firefighters navigating a burning building to identify and remove explosive hazards among visually similar distractors. The experiment assessed the effects of self-generated expectations of prevalence, instructed prevalence, and stress on high-stakes visual search.

On day one, participants were familiarized with the VE and task set up. Day two was divided into training and test stages: Participants were trained with an average of two or six targets (explosive hazards) and then tested with an average of two or six targets. Before testing, they received instructions accurately indicating whether target prevalence remained similar or changed across training and test stages. If training was the dominant influence, performance during the test should reflect their self-generated expectations:

• Participants trained with two targets were predicted to remove fewer false positives (i.e., removing distractors), and make more false negatives (i.e., misses) than those trained with six targets.
• Participants trained with six targets were predicted to remove more false positives, and have fewer false negatives than those trained with two hazards.

In contrast, if instructions had a greater influence, then these measures should reflect the instructions, which matched the number of targets presented at test; with high prevalence during the test resulting in fewer misses than low prevalence (cf. Wolfe et al., 2005, 2007).

Stress was manipulated within-subjects: Both training and test stages contained one low-stress and one high-stress session in a counterbalanced order. Low-stress sessions involved a quiet environment, a 2.5-min trial limit, and normal movement speed. High-stress sessions involved an ongoing alarm, explosion threats (which increased time pressure), and reduced movement speed. If high stress reduced the influence of self-generated expectations, error patterns in the high stress sessions should reflect the instructions received, whereas error patterns in the low stress sessions should reflect self-generated expectations.

## Methods

### Participants

Forty-eight participants (24 males, 24 females) were recruited from the student population of Cardiff University and received course credits or £25 for participating (mean age = 20.51 years, range = 18.17–21.59 years). Participants had normal or corrected-to-normal vision, did not wear prescription glasses, had no adverse experience with fire, and had not experienced motion sickness in the past three months; they were not currently ill, taking psychotropic medication, suffering from hypertension, or diagnosed with mood/anxiety disorders. Ethical clearance was granted by the School of Psychology Research and Ethics Committee (EC.22.04.26.6560GRA).

### Materials

**Figure 2.**
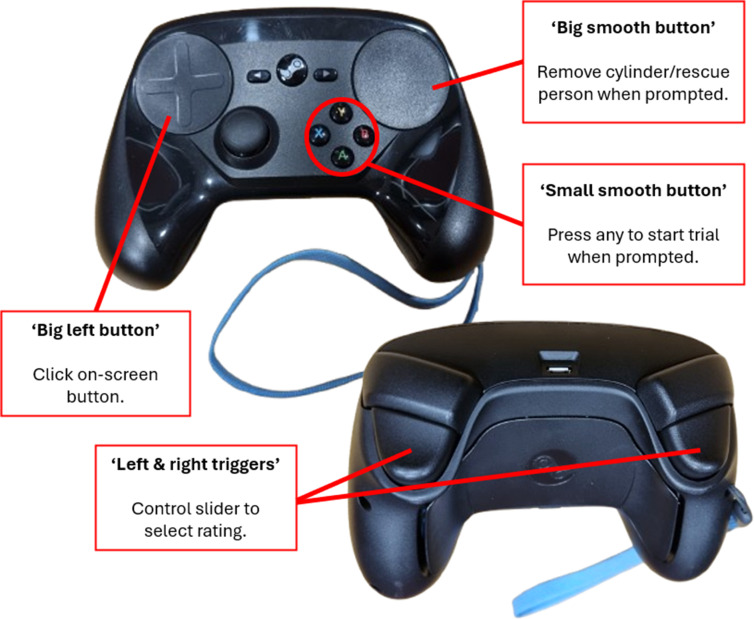
Button mappings on steam controller.

Figure 1 shows the 6-m diameter semi-immersive VE system (hereafter called the Igloo), where the experiment took place, and the Cyberith Virtualiser R&D Kit (omnidirectional motion platform controlling virtual locomotion). Appendix 1 details this setup. Participants interacted with the VE with the Steam Controller gamepad, with button mappings detailed in Figure 2.

#### Physiological Monitoring Apparatus

An emteqPRO Open Face Mask collected facial electromyograph (fEMG) data at 2000 Hz (Gnacek et al., 2022) to assess stress-induced facial expression changes. It has seven pairs of dry EMG electrodes and a photophlethysmogram (PPG) sensor, recording from the corrugator, frontalis, orbicularis oculi (hereafter called “orbicularis”), and zygomaticus muscles. Adjustable side inserts housing the orbicularis and zygomaticus electrodes ensure proper fit and signal quality across face widths (Gnacek et al., 2022).

A separate heart rate (HR) monitor was used due to reduced PPG signal quality of the emteqPRO mask during movement. The PolarOH1+, a 6-LED, arm-worn PPG HR monitor updates at 1 Hz and shows high intraclass correlation (0.95–0.99) with electrocardiogram-derived HR (Hettiarachchi et al., 2019).

### Hazard Search Task Design

The experiment was built by YST in Unity 2020.3.6f1 with the support of the Unity Experiment Framework (UXF; Brookes et al., 2020). Other Software Development Kits (SDKs) and plugins such as the EmteqVR SDK (emteq labs, 2022), Igloo Toolkit (Selly, 2022), OpenVR XR Plugin (SteamVR Unity Plugin, 2017/2023), and Cyberith Virtualiser SDK (Cyberith, 2020) were integrated to support the functionality of their associated equipment.

#### Building and Cylinders

**Figure 3.**
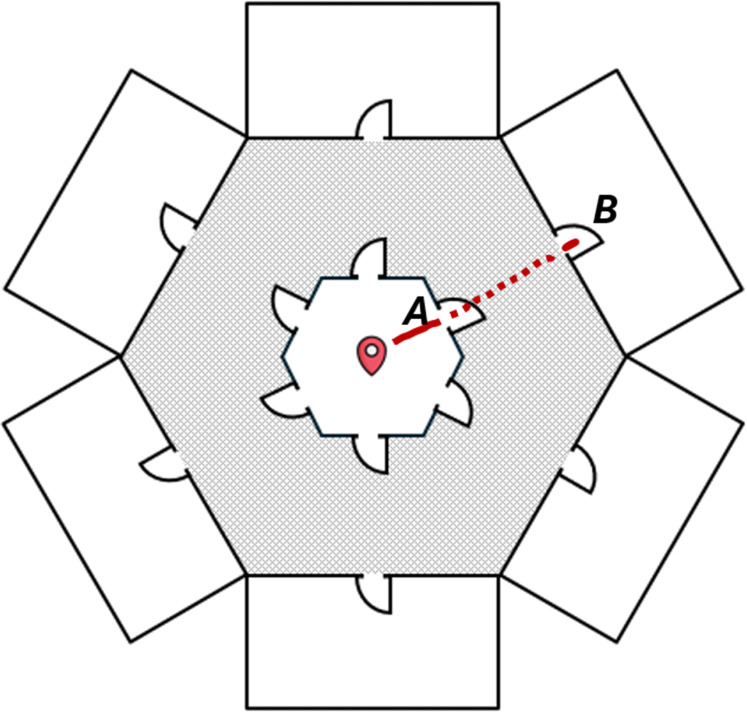
Ariel view of building layout. The red marker indicates the starting point in each trial. Doors in the smaller hexagonal area seamlessly teleported participants to the corresponding rooms to save time and reduce fatigue. The grayed-out area was invisible to participants. The solid and dotted red lines show a participant entering door A and getting teleported to location B.

The smoke-filled virtual building had six rooms in a circular arrangement (see Figure 3), with their inner walls forming a central hexagonal space where each trial began. A smaller hexagonal space with teleporting doors surrounded this starting point to reduce walking time and fatigue from covering large room-to-room distances. Entering the doors in the smaller hexagonal space teleported participants immediately and seamlessly to their corresponding rooms.

**Figure 4.**
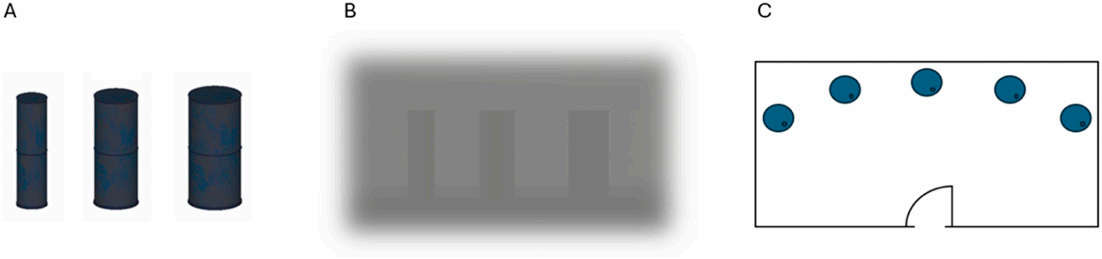
(a) From left: Small/easy distractor, target, large/difficult distractor. (b) The same stimuli in the building. (c) Ariel view of cylinders arranged in an arc within a room.

During the experiment, participants searched for middle-width cylinders (explosive hazards) amongst narrower and wider distractors within the building. Each of the first five rooms visited had five cylinders in an arc (Figure 4(c)), and the trapped person always appeared in the remaining room. In total, 25 cylinders of the same height (2.6 Unity-units) were shown in each trial. Based on pilot research, the target cylinders (targets) were 1.50 Unity-units wide; the small cylinders (easy distractors) were 30% narrower and the large cylinders (difficult distractors) were 20% wider (Figure 4(a) and (b)). While the building had at least one target, each room contained between zero and three targets. Each room had two cylinder types, allowing participants to compare sizes to identify the targets(s) without resorting to picking the middle-sized cylinder, which would be possible if all three types were present. Each trial included 12 easy distractors plus:

• Two-Targets condition (a mean of two targets per trial): One to three targets and 10–12 difficult distractors
• Six-Targets condition (a mean of six targets per trial): Five to seven targets and six to eight difficult distractors

#### The Hazard Search Task

**Figure 5.**
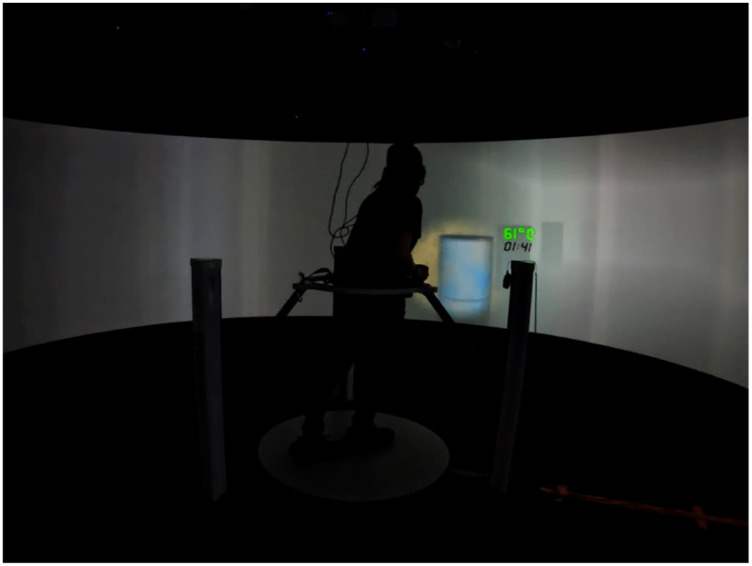
Virtual headtorch illuminating parts of a smoky room, virtual temperature reading, and countdown timer.

Participants began each trial at the starting point (see Figure 3). A virtual headtorch followed their head movements, while a virtual temperature reading and 2.5-min countdown timer followed it along the horizontal plane (see Figure 5). After pressing “a [small smooth button]” to start a trial as prompted, they visited each room to identify and remove targets. Doors opened automatically when participants were close and remained open. Upon entering a room, participants were immobilized for 2 s to discourage removing cylinders without due consideration. Participants removed a cylinder by approaching it until a prompt appeared (“Press the [big smooth button] to remove the cylinder.”) and pressed the specified button. If the removed cylinders were targets then they disappeared with a “ding!”, but if they were distractors they disappeared without a “ding!”. Moving away without pressing the button removed the prompt but not the cylinder. A similar prompt appeared when approaching the trapped person in the last room. When a trial ended, on-screen feedback indicated whether all targets were removed, the victim rescued, and both tasks completed in time. If both tasks were completed in time, then a congratulatory sound was played; otherwise, a failure sound was played.

Under High Stress, the temperature reading was red (90°C–95°C). Participants were informed that this, along with an ongoing alarm (73 dB on average), signaled a possible mid-trial explosion ending the trial unless all targets were removed in time. Unbeknownst to them, movement speed was approximately 40% slower than in the quiet (<50 dB), Low Stress condition, which had green temperature readings (60°C–65°C) signaling a low risk of explosion. Pilot research indicated that these manipulations of stress were effective using self-report and our physiological measures.

### Procedures

**Figure 6.**
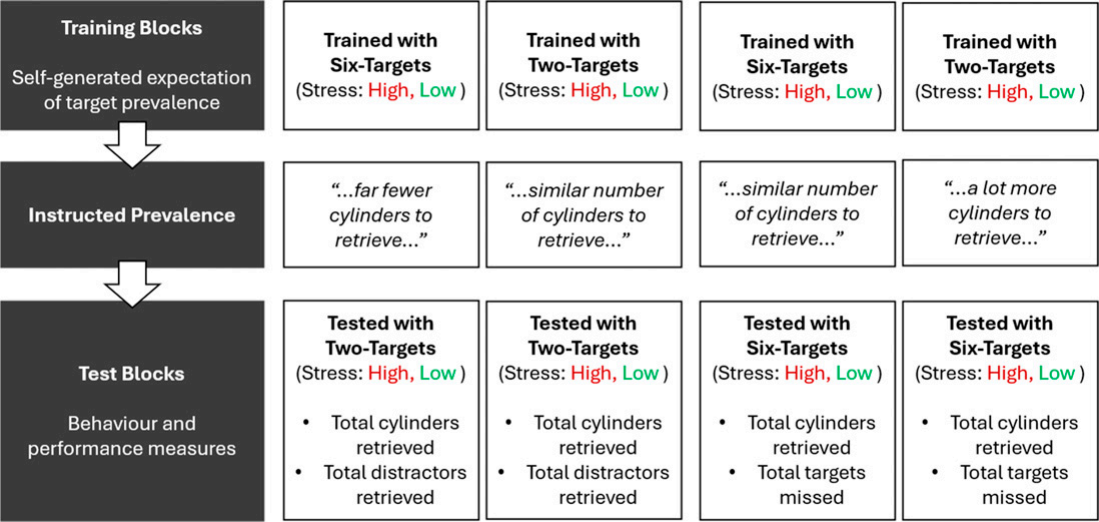
Schematic plan of the experimental design, showing the target prevalence in training and test blocks, and the instructed prevalence for each condition. The order of high and low stress blocks was counterbalanced.

Stress was manipulated within-subjects during training and test. Target prevalence during training and test was manipulated between-subjects using a 2 × 2 factorial design (two or six targets on average during training and during test; see Figure 6). Day One involved acclimation to the VE and learning the game mechanics, and Day Two (the next day) included training and test sessions.

#### Day One: Acclimation and Learning

Day One sessions were restricted to 1 h. Participants were equipped with an emteqPRO mask, a PolarOH1+ on their bicep, a Vive Tracker on their head, and low-friction overshoes from Cyberith GmbH. The researcher provided safety instructions, assisted participants onto the motion platform, secured them in place, and guided them in walking using video demonstrations and verbal instructions. They practiced walking in a virtual park environment for up to 20 min and learned to use the Steam Controller (see Figure 2). A three-minute mask calibration followed, the data from which was not analyzed.

**Figure 7.**
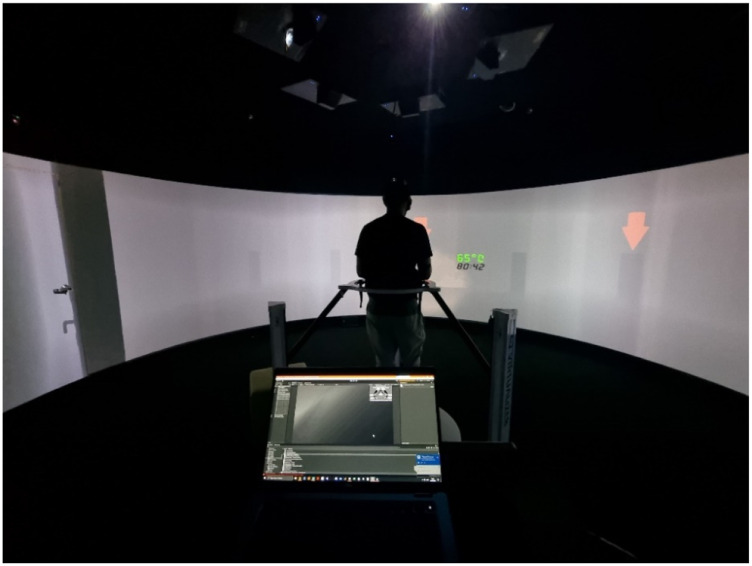
Orange arrows marking targets during the training phase.

All trials were self-paced. Participants were briefed that they would be acting as firefighters, learning to identify target cylinders amongst similarly sized distractors, with a 2.5-min limit per trial applying only in the experiment proper. A passive and an active training block each had four trials under Low Stress, with target prevalence matching the experimental condition participants were assigned to. This training task resembled the hazard search task with some exceptions: During passive learning trials, participants observed the cylinders from the doorway without entering the rooms. They were informed that after 4 s, orange arrows would mark targets with a “ding!” and that only cylinders of similar width should be removed to prevent explosions (Figure 7). No arrows or “ding!” after the wait indicated no targets were present. Participants were reminded to learn the identity of the hazardous cylinders by comparing cylinder sizes before leaving for another room, because they would need to identify them unaided in the active learning trials. These instructions were given during a guided demonstration of the first passive learning trial.

After four trials and an optional break, the active learning trials started. These trials were identical to the hazard search task but untimed. Two additional High Stress trials followed; the researcher described their features and warned that explosions, while currently absent, would happen randomly during the main experiment. Participants then completed the first trial. Performance feedback was provided after each active learning trial. In the second trial, they were warned before an explosion was manually triggered. After confirming their comfort with this level of stimulation for the experiment proper (on Day Two), participants left.

#### Day Two: Experiment

Day Two sessions were restricted to 1.5 h. Participants were equipped with the same equipment. They practiced walking on the motion platform to regain familiarity. Then, they stood still, taking deep breaths for 2 min to record baseline HR. For mask calibration, they displayed maximum expressions (smile, frown, raised eyebrows), each alternating with a neutral expression. They were re-briefed about the experiment, task, 2.5-min time limit, and stress conditions, and informed that the researcher would only enter the Igloo during breaks for checks and readjustments. The experiment began once the researcher left the Igloo and signaled the participant to start.

Participants first received two training sessions, which each included four trials. Half of the participants received one to three targets (i.e., Two-targets training) and the remainder received five to seven targets (i.e., Six-targets training; see Figure 6). The order of the Low and High Stress sessions was counterbalanced, with two trials exploding 90 ± 2.5 s in. Each of the two test sessions contained three trials. Among participants who trained with six targets, half were tested with six targets and half with two. Similarly, among those trained with two targets, half were tested with six targets and half with two. For a given participant, the order of the Low and High Stress sessions matched the order during training, with an explosion in the second trial. Performance feedback was provided after each training trial to shape prevalence expectations, but not during test trials. During training and test, a three-minute break (extendable upon request) separated each session.

At the start of testing, participants received the Instructed Prevalence: “Before you start, I need you to know that there is/are ___ to remove in the building now as/than there were before.” When training and test prevalence matched, the blank was replaced with “a similar number of cylinders”; otherwise, it was “a lot more cylinders” or “far fewer cylinders” when prevalence increased or reduced, respectively. After each session, participants were asked “How were you coping with the search and rescue tasks just now?”, from 1 (I felt no pressure) to 10 (I was unable to cope with the pressure).

##### Response Measures for the Hazard Search Task

The effects of Training Prevalence (self-generated expectations of prevalence) and Instructed Prevalence on search performance were assessed with the number of false positives (distractors removed) and false negatives (targets missed). Hits (targets correctly removed) were not analyzed as they are the inverse of false negatives and provide no additional information. For a given test prevalence (e.g., six targets), similar search performance across training prevalence (six or two targets) would suggest an influence of Instructed Prevalence or actual prevalence during the test. Whereas if search performance was affected by Training Prevalence, it would suggest that the expectations generated by training were influential.

##### Data Preparation

The emteqPRO recordings generated a CSV file, from which mean amplitudes (μV) for each session were derived with a modified Python script from Emteq Labs (emteq labs & Mavridou, 2025). Five-second periods of explosions were excluded to reduce bias from abrupt facial expressions, and mean amplitudes from bilateral muscles (frontalis, orbicularis, zygomaticus) were averaged by muscle group. Mean HR (bpm) for each session was calculated from the PolarOH1+ CSV file using another Python script, accounting for a 17-s lag (identified in prior testing) and the same 5-s explosion periods. Session means were computed for false positives and false negatives. Trials where participants failed to rescue the victim were excluded from this analysis. All statistical tests were performed using R, version 4.4.0 (R Core Team, 2024). Where assumptions for parametric tests were violated, non-parametric alternatives were conducted or robust methods used (Maechler et al., 2006; Mair & Wilcox, 2020).

## Results

When trials did not end prematurely with an explosion, most participants rescued the trapped person within the allotted time (median = 100%, IQR = 8%), indicating high levels of task engagement. On Day One, total passive learning duration was similar across the Two-Targets (median = 612.36 s, IQR = 105.78 s) and Six-Targets groups (median = 590.36 s, IQR = 125.56 s), Wilcoxon signed-rank test, *W* = 317, *p* = .56. The Two-Targets group (median = 762.13 s, IQR = 212.79 s) completed all active learning trials significantly quicker than the Six-Targets group (median = 899.43 s, IQR = 286.47 s), *W* = 176, *p* = .02. Table 1 shows similar median test durations across groups on Day Two, Kruskal–Wallis test, X^2^ (3) = 3.65, *p* = .301.

**Table 1.** Median (IQR) Test Duration on Day Two.

Tested with two targets	
Trained with six targets	2267.39 s (428.08 s)
Trained with two targets	2283.59 s (515.53 s)
Tested with six targets	
Trained with six targets	2399.83 s (140.27 s)
Trained with two targets	2282.89 s (258.02 s)

### Stress Measures

Paired t-test showed that mean perceived difficulty coping was significantly lower in the low stress sessions (mean = 2.94, SE = 0.21) than the high stress sessions (mean = 4.91, SE = 0.26), *t* (47) = 9.82, *p* < .001, Cohen’s *D* = 1.42. Due to technical issues, fEMG data was missing from two participants. Table 2 shows that median amplitudes (μV) were similar across stress conditions for each muscle group (*n* = 46), as confirmed by Mann–Whitney tests: corrugator (*Z* = 0.91, *p* = .37), frontalis (*Z* = 0.42, *p* = .67), orbicularis (*Z =* 1.02, *p* = .31), and zygomaticus muscles (*Z* = 0.50, *p* = .62). Average HR did not differ under low (median = 109.85 bpm, IQ = 14.75 bpm) and high stress (median = 111.84 bpm, IQR = 15.65 bpm), *Z* = 1.06, *p* = .288.

**Table 2.** Median (IQR) Facial Muscle Amplitudes (μV) in Low and High Stress Conditions.

	Low Stress	High Stress
Muscle group		
Corrugator	3.51 (1.85)	3.98 (2.17)
Frontalis	6.91 (5.09)	7.02 (6.19)
Orbicularis	4.24 (2.35)	4.96 (2.43)
Zygomaticus	6.35 (14.78)	7.08 (11.16)

### Test Blocks: False Positives

**Figure 8.**
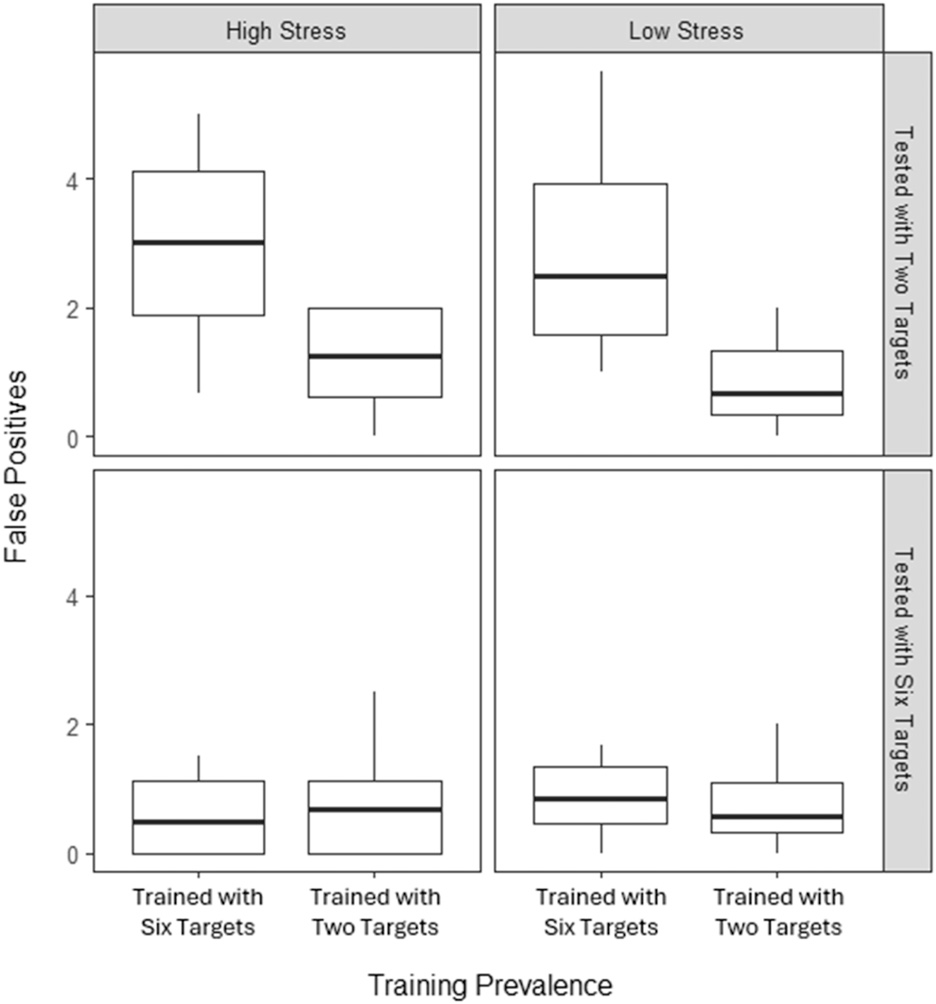
Boxplot including the median number (bold line) and interquartile range (box) of false positives in the test in both stress conditions, separated by whether training and test involved two or six targets.

Figure 8 shows that those trained with six targets made more false positives when tested with two targets, than those trained with two targets (upper panels). No such difference appeared for those tested with six targets (lower panels). This pattern of results was evident in both high stress (left panels) or low stress (right panels) sessions. A mixed ANOVA confirmed significant main effects of training prevalence (*F* (1, 44) = 12.98, *p* = .001, η^2^_G_ = .20) and test prevalence (*F* (1, 44) = 22.22, *p* < .001, η^2^_G_ = .30), but no effect of stress (*F* (1, 44) = 0.37, *p* = .546, η^2^_G_ = .00). There was also a significant interaction effect between training and test prevalence (*F* (1, 44) = 12.86, *p* = .001, η^2^_G_ = .20). Simple effects analyses confirmed a significant difference between the groups tested with two targets (*p* < .001), but not six targets (*p* = .987). No other interactions were significant (*p*s = .093–.920).

**Figure 9.**
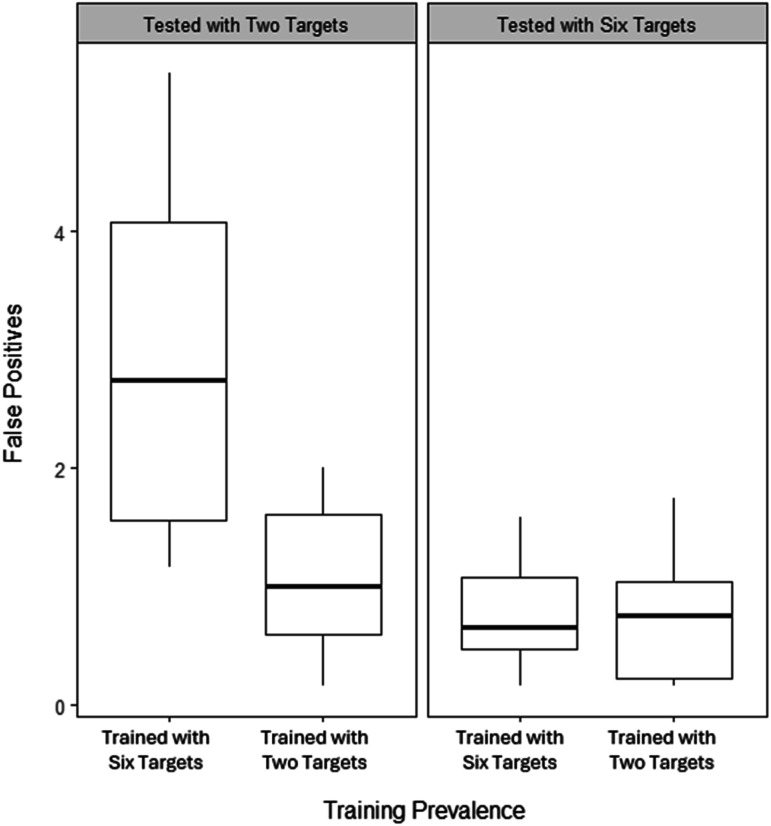
Boxplot showing the median number (bold line) and interquartile range (box) of false positives in the test by training and test prevalence.

Due to heterogenous variances and nonnormality, a robust between-subjects ANOVA was conducted collapsed across stress conditions. Figure 9 shows average false positives by groups. There were significant main effects of training prevalence (*X*^*2*^ = 7.50, *p* = .016) and test prevalence (*X*^*2*^ = 14.63, *p* = .002), and a significant interaction between them (*X*^*2*^ = 6.83, *p* = .020). Robust independent t-tests confirmed a significant difference between those tested with two targets (*F* (1, 9.43) = 8.26, Bonferroni-corrected *p* = .035, ξ = 0.77), but no significant difference between those tested with six targets (*F* (1, 12.19) = 0.03, Bonferroni-corrected *p* = 1.00, ξ = .05).

### Test Blocks: False Negatives

**Figure 10.**
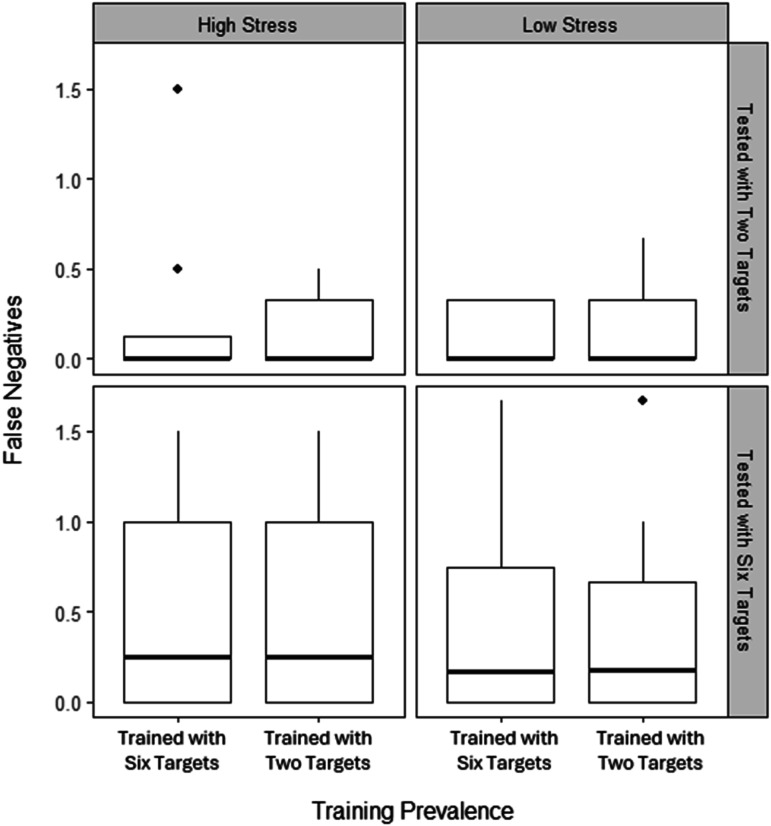
Boxplot including the median number (bold line) and interquartile range (box) of false negatives in the test across stress conditions, separated by whether training and test involved two or six targets. Individual points indicate outliers.

Figure 10 shows slightly fewer false negatives when tested with two targets than with six, but no effect of training prevalence or session type (high or low stress). Mixed ANOVA confirmed a significant main effect of test prevalence, (*F* (1, 44) = 5.81, *p* = .020, η^2^_G_ = .08), but not other main or interaction effects (*p*s = .297–.998).

**Figure 11.**
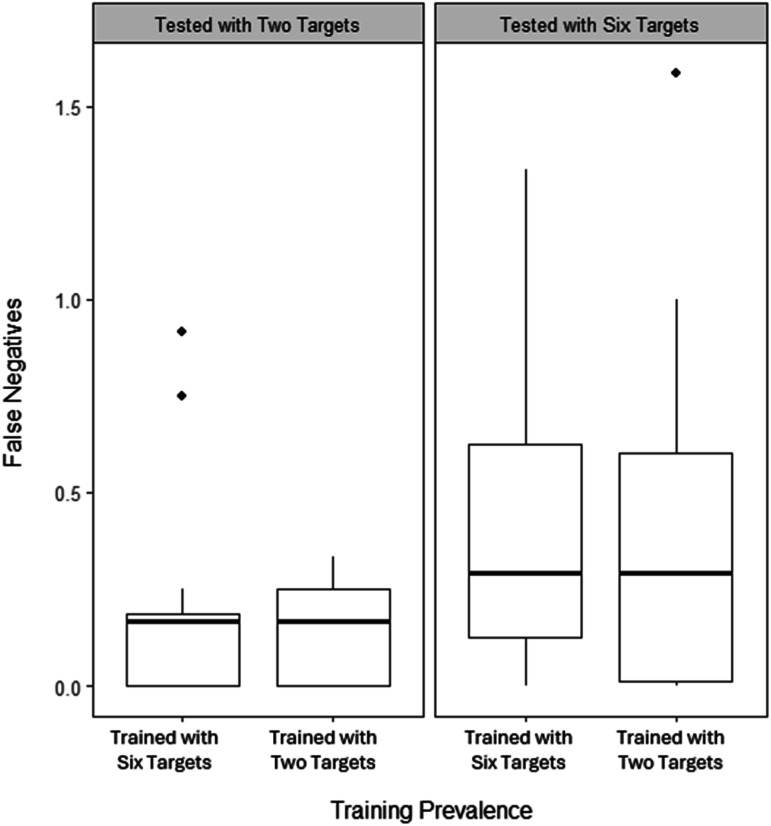
Boxplot showing the median number (bold line) and interquartile range (box) of false negatives depending on whether training and test involved two or six targets. Individual points indicate outliers.

Due to heterogenous variances and nonnormality, a robust between-subjects ANOVA was conducted, collapsing across stress condition. Figure 11 shows average false negatives across conditions. The main effects and interaction were not significant (test prevalence: *X*^*2*^ = 3.73, *p* = .071; training prevalence: *X*^*2*^ = 0.03, *p* = .865; interaction: *X*^*2*^ = 0.07, *p* = .789).

## Discussion

The present study investigated how self-generated and instructed expectations about target prevalence affects visual search in high-stress, high-stakes scenarios. In a simulated search and rescue mission, participants identified and removed explosive hazards amongst visually similar distractors, before rescuing a trapped person. Under high stress, an alarm, the risk of trial-ending explosions, and artificially reduced movement speed created time pressure during visual search. Under low stress, these stressors were absent. After the first two training blocks, which established self-generated prevalence expectations, participants were informed that target prevalence would change or remain the same in the two test blocks. Visual search during the test was jointly influenced by training and test prevalence: When test prevalence was low, those trained with a similar number of targets made fewer false positives than those trained with more targets. When prevalence was high, training prevalence had no effect on false positives. False negatives were low across conditions (Figure 10), indicating that all targets were removed most of the time. Perceived high stress levels did not alter the pattern of findings. These findings are consistent with prior research demonstrating the dominance of self-generated prevalence expectations over instructed prevalence in high-stakes visual search (Ishibashi et al., 2012; Experiments 1a and 1b, Ishibashi & Kita, 2014; Lau & Huang, 2010).

Our stress manipulation increased self-reported but not physiological markers of stress. A prior pilot study used the same stressors in a similar environment found significantly greater facial expressiveness under high stress (see Dinges et al., 2005), reflected in elevated fEMG amplitudes across the zygomaticus, orbicularis, frontalis, and corrugator. The absence of such effects in the present study likely reflects additional visual search demands (see Anderson, 2024; Anderson & Lee, 2023; Attar et al., 2016; see also, Berger et al., 2020), potentially masking stress-related facial activity observed under the task of simply locating a person in the pilot. Similarly, exertion from using the motion platform might have masked stress-related HR changes, a pattern consistent with the pilot study that employed the same setup. Finally, the stressors might have been too mild to elicit robust physiological responses here, as they were designed to induce stress without overwhelming participants in a confined space.

The set-up of the present study precluded the derivation of sensitivity (d’) and criterion (C) measures, as it bore similarities to multi-target searches (e.g., Cain et al., 2014). Given comparable false negatives across groups, differences in false positives provide a proxy measure of changes in criterion, with more false positives indicating a more liberal decision threshold. Therefore, learned decision thresholds likely failed to adapt quickly to the instructed prevalence when test prevalence was low, which is consistent with Lau and Huang’s (2010) description of visual search performance as being governed mostly by prior experience. When test prevalence was high, the influence of self-generated prevalence was not evident. However, low false negatives across groups complicate the interpretation of this null effect under high test prevalence: The influence of self-generated expectations could be present but obscured by floor effects. Alternatively, if self-generated expectations truly had no influence, this could instead suggest a conditional ability to inhibit their influence, such as when high target prevalence encourages reliance on perceptual cues over prior expectations.

The influence of training prevalence on test performance with low target prevalence aligns with the findings of Hon and Jabar (2018). They investigated the amount of exposure needed to learn and apply prevalence information during visual search. Using reaction time (RT) to index prevalence effects (e.g., longer RTs at low prevalence), they found that when prevalence changed halfway through a 600-trial block, RT adjusted to the new prevalence within 10 target trials (whether targets appeared close together or spaced apart) and stabilized thereafter. It was proposed that prevalence information was incorporated into task representations within those initial trials and influenced subsequent performance (i.e., RTs) consistently (Hon & Jabar, 2018). Similarly, in the present study, prevalence expectations acquired during training continued to bias search performance despite explicit instructions about changes in prevalence. Instructed prevalence, while accurate but not yet experienced, likely required time to recalibrate task representations to reliably debias search performance. Moreover, the lack of corrective feedback (i.e., whether all targets were removed) at the end of each test trial might have reinforced initial biases (Cox et al., 2021; Wolfe, 2022) and further delayed this process.

The observation that training prevalence influenced test performance even under (perceived) high stress suggests that any resulting depletion of cognitive resources did not disrupt self-generated prevalence expectations during the test (cf. Kemper et al., 2012). In visual search, self-generated expectations may operate differently from or rely less on the same attentional processes implicated in self-generated expectations during other visual tasks, such as shape discrimination (e.g., Gaschler et al., 2014; Kemper et al., 2012).

### Low Prevalence Did Not Increase False Negatives

In single-target searches, the low prevalence effect (LPE) refers to a strong tendency to miss targets on rarely-occurring target-present trials interleaved with target-absent trials (Wolfe et al., 2005, 2007). Interestingly, the LPE, or an analogous effect, was not observed here: False negatives during the test remained similar regardless of training or instructed prevalence. In the present study where all trials were target-present, low prevalence was instead defined by relatively fewer targets per trial. As participants were never exposed to target-absent trials, the present procedure was not directly analogous to standard visual search procedures where the LPE was observed.

The present findings could also result from task framing. Although speed *and* accuracy were both emphasized to participants, missing targets (i.e., explosive hazards) were more consequential than the limited time costs of removing a distractor, provided the trials were completed in time. It would be fruitful to investigate whether the dominance of training prevalence would persist when false positives and false negatives carry similar consequences. Moreover, visual search performance and task performance did not fully overlap: Successfully rescuing the trapped person required the timely removal of all targets, whereas false positives carried no penalties that would affect the rescue beyond a slight delay, at least under low stress conditions. In other words, adopting a more cautious approach might compromise search performance but not task success. Whether these strategic considerations shaped participants’ behavior, including their reliance on training prevalence (under low test prevalence), is unknown.

Our results demonstrate that in high-stakes contexts, like firefighting and emergency response, visual search is affected by previously learned information, even when new and accurate prevalence information is provided. Specifically, those trained under high prevalence made more false positives when instructed that prevalence would greatly decrease, likely reflecting difficulties in adjusting their decision criteria rapidly. In real-world contexts, this might translate into time and effort being misdirected to innocuous stimuli misidentified as threats, potentially delaying response to genuine threats and increasing the likelihood of adverse outcomes in time-sensitive situations. The persistence of this effect under high stress, time-pressured conditions suggests that the influence of self-generated expectations might operate in a way that is relatively immune to cognitive resource depletion, rather than easily replaced by new information. These findings have important implications for visual search in first responders, suggesting that mission-critical information communicated in plain language might not enhance search performance if it contradicts previous experience.

The use of student participants here investigated the influence of self-generated and instructed prevalence expectations in an untrained population, providing a baseline for understanding how experience and training shape visual search in high-pressure domains. However, it should be acknowledged that with *n* = 12 for between-subjects and *n* = 24 for within-subjects comparisons, the study may have been underpowered to detect more subtle differences. Future work could extend this research by replicating it with larger samples and increasing task difficulty to address ceiling effects: For example, by identifying the just-noticeable difference between targets and distractors for each participant and adjusting the stimuli accordingly. The stress manipulation could be further enhanced by introducing a social evaluative component in which participants are led to believe that their performance is recorded and evaluated. Future research might also investigate how reliance on established target prevalence could be overcome. For example, one possibility would be to regularly alter target prevalence during training exercises, as previous work shows that the influence of self-generated prevalence expectations can be reduced under such conditions (see Schwager et al., 2017; see also, Kramer et al., 2019). Implementing such VR-based training for first responders could improve adaptability under pressure. Understanding how to reduce the influence of self-generated expectations is one way in which visual search could be improved during emergencies and enable the development of more effective training protocols and communication strategies.

## Key Points

• High-stakes visual search under stressful conditions could be guided by prior expectations or new information about target prevalence. Here, participants identified explosive hazards among visually similar distractors in a semi-immersive virtual environment, guided either by expectations formed during training or newly provided prevalence information.
• Irrespective of differences in self-reported stress levels, visual search performance remained biased by prior expectations when the number of targets at test was low, but not when the number of targets was higher.
• These results emphasize the value of integrating techniques and tools into training programs that help first responders counteract the influence of outdated probabilistic information during high-pressure visual searches.

## Data Availability

Data and analyses are available on Open Science Framework: https://osf.io/cagfp/overview.

## Appendix

### Appendix 1

#### Virtual Environment (VE) Setup

The experiment took place in an Igloo Immersive Cylinder (hereafter called the Igloo), a 6-m diameter semi-immersive VE system from Igloo Vision Ltd. The Igloo incorporated five Epson EH-LS500B projectors on its ceiling, which enabled the projection of 360° content onto a cylindrical screen (Figure 1(a)). A surround sound system supported immersive audio with five Cambridge Audio MINX MIN12 loudspeakers fixed to the circular frame inside and at the top of the cylinder, a Cambridge Audio SX-120 subwoofer outside of the frame, and a Denon AVR-X550BT amplifier. All audio was played at 70% of the maximum amplifier volume, and the sound level in different experimental conditions were measured with the Radio Shack Digital Sound Level Meter (model 33-2055): 73 dB on average in the High Stress condition, and below 50 dB in the Low Stress condition. Three SteamVR Base Station 2.0, mounted on the circular frame, supported communication between the VIVE Tracker 3.0, VIVE Pro HMD, and related software. Two 10-m-long active USB extender cables hung down from the top of the cylinder, connecting the emteqPRO mask to the main computer next to the Igloo. A portable Tripp Lite air-conditioning unit was installed for user comfort.

To increase realism, locomotion in the virtual environment was controlled by an omnidirectional motion platform in the middle of the Igloo: The Cyberith Virtualiser R&D Kit. It had a low-friction, circular baseplate, and a ring that secured the participant in place. The participant wore low-friction overshoes and got onto the platform when the safety ring was lowered from its usual position. The ring was then lifted to the participants’ hips and locked with three pins on the pillars to prevent slips and falls. While the researcher tightened the harness, participants were told to keep the adjustment plunger in front of their belly button to ensure correct heading (Figure 1(b)). Six optical motion sensors in the baseplate recorded movement speed at 1000 Hz, while one in the ring tracked rotation, translating movement data into the virtual world for a near-natural walking experience. Movement speed could also be increased or decreased by adding a movement speed multiplier to the “CVirtPlayerController” script component (Effective VR Treadmill - Cyberith Virtualizer R&D Kit, nd).
